# Triflamides and Triflimides: Synthesis and Applications

**DOI:** 10.3390/molecules27165201

**Published:** 2022-08-15

**Authors:** Mikhail Y. Moskalik, Vera V. Astakhova

**Affiliations:** Irkutsk Institute of Chemistry, Siberian Branch of the Russian Academy of Sciences, 664033 Irkutsk, Russia

**Keywords:** triflamide, triflimide, amination, heterocyclization, NH-acids, sulfonamidation, coupling, biological activity

## Abstract

Among the variety of sulfonamides, triflamides (CF_3_SO_2_NHR, TfNHR) occupy a special position in organic chemistry. Triflamides are widely used as reagents, efficient catalysts or additives in numerous reactions. The reasons for the widespread use of these compounds are their high NH-acidity, lipophilicity, catalytic activity and specific chemical properties. Their strong electron-withdrawing properties and low nucleophilicity, combined with their high NH-acidity, makes it possible to use triflamides in a vast variety of organic reactions. This review is devoted to the synthesis and use of *N*-trifluoromethanesulfonyl derivatives in organic chemistry, medicine, biochemistry, catalysis and agriculture. Part of the work is a review of areas and examples of the use of bis(trifluoromethanesulfonyl)imide (triflimide, (CF_3_SO_2_)_2_NH, Tf_2_NH). Being one of the strongest NH-acids, triflimide, and especially its salts, are widely used as catalysts in cycloaddition reactions, Friedel–Crafts reactions, condensation reactions, heterocyclization and many others. Triflamides act as a source of nitrogen in C-amination (sulfonamidation) reactions, the products of which are useful building blocks in organic synthesis, catalysts and ligands in metal complex catalysis, and have found applications in medicine. The addition reactions of triflamide in the presence of oxidizing agents to alkenes and dienes are considered separately.

## 1. Introduction

Over the past 2–3 decades, *N*-Trifluoromethanesulfonamides (CF_3_SO_2_NHR, TfNHR) have found wide application in organic synthesis as reagents, catalysts, additives and as substituents that transform reactivity and biological activity in a wide range of substrates. The chemistry of triflamide and triflimide derivatives was the subject of several early reviews [1,2], which confirms the high interest in such compounds. Having a strong electron-withdrawing CF_3_SO_2-_group in their structure, triflamides are among the strongest NH-acids (*pKa* (in H_2_O) for TfNH_2_ is 6.33, *pKa* (in H_2_O) for Tf_2_NH is 2.8 [1]). This property determines the use of triflamides in organic synthesis, in the production of biologically and pharmacologically active substances and in various industries. One of the most important properties of triflamide derivatives in terms of biological activity is their lipophilicity. Triflamides are widely used in the production of lithium–sulfur batteries, where *N*,*N-*dialkyl-substituted triflamide (dimethyl- or dipropyl-) is present as a solvent in the electrolyte [3]. In organic synthesis, catalysts containing a trifluoromethanesulfonamide or α-imide moiety are used (Michael [4], Friedel–Crafts [5], Diels–Alder [6], Mannich [7] and many other reactions).

This review is devoted to the latest progress in the field of applications of triflimide in organic synthesis as an active catalyst. The review also includes the reactions of triflimide and its derivatives (metal salts). As a catalyst/co-catalyst based on the Tf_2_N-salts of metals (Au, Ag, Fe, Li, Ca), triflimide has found application in a wide range of addition reactions, cycloaddition, intramolecular cyclization, CH-amidation, etc. Tf_2_NH is often used as an additive to the reaction medium, for example, in the synthesis of spiroheteropolycyclic compounds [8,9] and nitrogen-containing heterocycles [10], in catalytic (3 + 2)-annelation [11], in the oxidative synthesis of hydrodibenzofurans [12] and in condensed 2,8-*O*,*O*- or *O*,*N*-bicyclo[3.3.1]nonanes [13]. The triflimide anion is a counterion for the production of low-melting ionic liquids, used to stabilize nanoparticles, which are used in various fields, including medicine, sensors, optics and the aerospace industry [14,15]. On the basis of triflimide, various types of extractants and ionic liquids with organic cations have been obtained, which are used to isolate lanthanides and actinides from liquid waste; for example, from spent nuclear fuel [16,17].

The triflamide moiety is introduced in organic molecules in two ways. The first way is the reaction of a substrate with the activated TfNHR sulfonamide molecule. The second method is the treatment with trifluoromethanesulfonic acid anhydride or halides of the corresponding *N-*nucleophiles, which, as a rule, require low temperatures and the presence of additional bases.

## 2. Triflimide as a Catalyst in Organic Synthesis

Triflimide **1** (Tf_2_NH) is used as a catalytic additive in the formation of C-C and C-heteroatom bonds, due to its strong acidity, as well as its good compatibility with various organic solvents [18]. It has been widely used as a Brønsted acid for the catalysis of Friedel–Crafts reactions [19,20,21,22,23,24] and cycloaddition reactions [25,26]. In addition, triflimide is used as a Brønsted acid, for example, to obtain various bis-arylated amides from vinyl azides in moderate to quantitative yields [27] or in the hydroalkylation of arylalkenes with activated alcohols [28]. In a series of works, Ye et al. proposed different approaches to the cyclization of ynamides initiated by Tf_2_NH, which were applied to the synthesis of functionalized heterocyclic compounds [29,30,31,32,33].

**Scheme 1:**
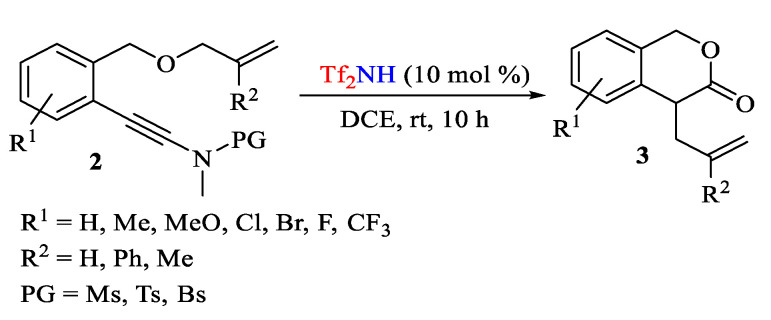
Metal-free intramolecular alkoxylation-initiated cascade cyclization in the presence of Tf_2_NH.

**Scheme 2:**
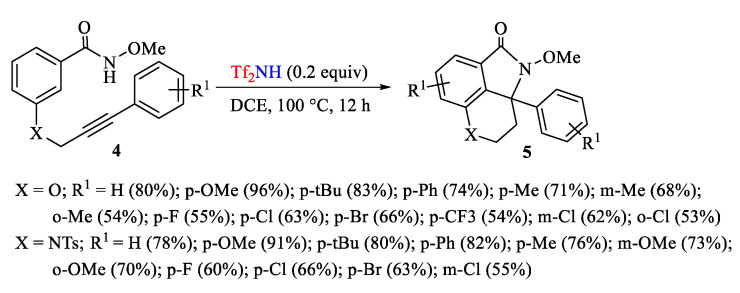
Intramolecular cyclization of *N-*methoxybenzamides in the presence of Tf_2_NH.

For example, the cascade cyclization of ynamides **2** having an allyl ether moiety in the molecule made it possible to obtain various highly functionalized 3-isochromanones **3** via intramolecular alkoxylation in good to quantitative yields (68–99%) under mild conditions (Scheme 1) [30]:

Based on these works, it was proposed to activate simple alkynes and to synthesize a number of highly functionalized isoindolinones [34]. The intramolecular Tf_2_NH-initiated cascade cyclization of *N-*methoxybenzamides **4** gives rise to the formation of fused chromanes or isoindolinones **5** in good yields and with a high regioselectivity (Scheme 2). The reaction meets the requirements of green chemistry by providing an atom-economical alternative to transition metal catalysis.

Frontier et al. [35] described a simple intermolecular reaction of the carboamination of alkynols **6** with cyclic aminals **7** in the absence of metal-based catalysts. Acidic conditions (Tf_2_NH) allowed it to stereoselectively synthesize ether-condensed cyclic enamines **8**.

**Scheme 3:**
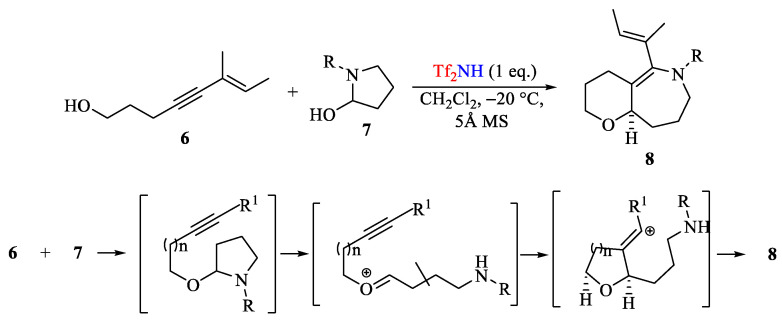
One-pot synthesis of bicyclic heterocycles by the reaction of carboamination of alkynols with cyclic aminals using metal-free approach.

**Scheme 4:**
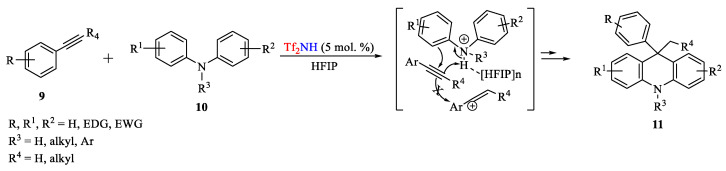
One-pot assembly of 9,10-dihydroacridine scaffolds.

The proposed reaction mechanism involves the formation of aminals by combining alkynols with aminals via the capture of iminium ions. Aminals give oxonium ions and vinyl cations, respectively, through the ring opening and subsequent reaction with alkyne. Finally, the intramolecular amination leads to the formation of cyclic enamines. Various bicyclic heterocycles were obtained in a good yield and with good diastereoselectivity (Scheme 3):

The one-pot assembling of the 9,10-dihydroacridine scaffolds **11** used in OLED devices was achieved by the selective *ortho*-C-alkenylation of diarylamines **10** with arylalkynes **9** followed by the intramolecular hydroarylation of the olefin formed as an intermediate. The reaction was carried out in hexafluoroisopropanol with triflimide as a catalyst which launched the reaction (Scheme 4) [36]:

Arylalkynes with electron-donating groups in the *para*-position give 9,10-substituted dihydroacridines in a 73 to 98% yield. Noteworthy, in the case of the nitrile and nitro groups, a complete conversion of the starting alkyne is achieved only at high temperatures of the reaction mixture. The effect of the diarylamine structure on the course of the reaction was also investigated, and it was shown that the presence of electron-donating or electron-withdrawing groups only slightly affected the reaction, as the target products were obtained in 57–95% yields.

**Scheme 5:**
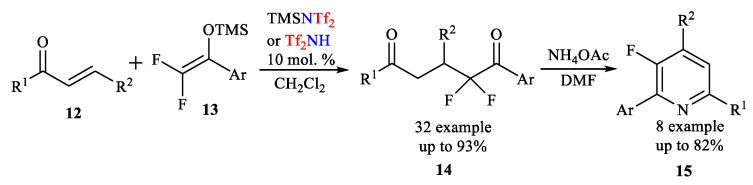
Difluoroalkylation of α,β-enones in the presence of TMSNTf_2_ or Tf_2_NH.

An efficient method was developed for the nucleophilic introduction of a difluorinated carbanion (from **13**) into α,β-enones **12** with the formation of 2,2-difluoro-1,5-diketones **14**, the regioselectivity of the reaction being determined by the TMSNTf_2_ (silylbistriflimide) or Tf_2_NH **1** catalyst. It is the strong electron-withdrawing properties and bulky substituents in the TMSNTf_2_ generated in situ that were responsible for the 1,4-addition route. 2,4,6-Triaryl-substituted 3-fluoropyridines **15** can be obtained by the one-pot method (Scheme 5) [37]:

However, with other bulky catalysts, such as trimethylsilyl trifluoromethylsulfonate (TMSOTf) or *tert*-butyldimethylsilyl trifluoromethanesulfonate (TBSOTf), which are also effective in this reaction, 1,2-addition by-products are also formed as minor admixtures. The electronic effects of the substituents in α,β-enone do not affect the yield of the products. The yield is slightly reduced in the presence of bulky substituents.

Triflimide is used in some cyclization in Diels–Alder reactions of 4-oxopent-2-enoates [38], in Michael addition reactions to substitute 3-vinyl-1,2,4-triazines and the subsequent cyclization to tetrahydro-[1,6]-naphthyridines [39] and in the synthesis of 2,3-dihydro-1*H*-benzo[*e*]indoles and 2,3-dihydrobenzofurans using Tf_2_NH. The yield of the latter was 64% (with Tf_2_NH) but while using In(OTf)_3_, the yield increased to 80% [40]. The synthesis of mono- and bis-γ-lactams by the Mannich method (the addition of 2,5-bis(trimethylsilyloxy)furan to imines) occurs in good yields in the presence triflimide [41]. The intramolecular hydroacyloxylation of non-activated alkenes proceeds well in hexafluoroisopropanol not only with triflimide, but also in the presence of Ca(NTf_2_)_2_+*n*Bu_4_NPF_6_ [42]. The aromatization of dibenzonorcaradienes to dibenzo[*f,h*]isocoumarins proceeds with TfOH in yields up to 99%. Replacing the triflic acid with triflimide also showed excellent results, affording the products in a 95–98% yield [43]. Enantioselective (4 + 2)-cycloaddition in the presence of triflimide gives chiral 1,2-amino alcohols, 1,2-diamines and β-amino acids in yields of 92–98% [44].

**Scheme 6:**
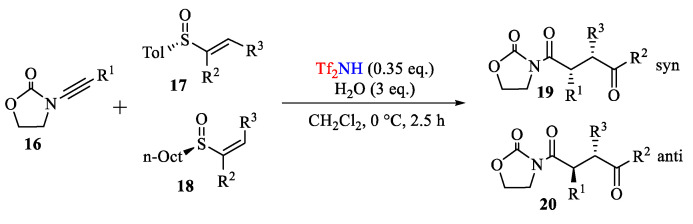
Reaction of vinyl sulfoxides with ynamides in the presence of Tf_2_NH in water.

**Scheme 7:**
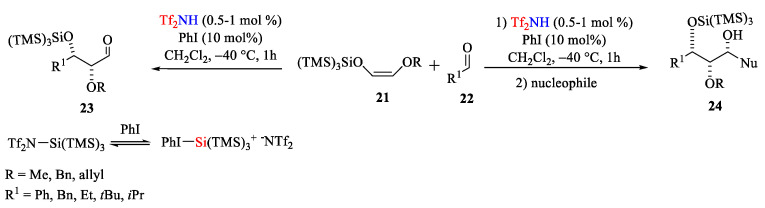
Aldol reaction of (Z)-tris(trimethylsilyl)silyl enol ethers in the presence of triflimide.

The Tf_2_NH-catalyzed (3,3)-sulfonium rearrangement of vinyl sulfoxides **17** or **18** in the presence of ynamides **16** is an enantioselective and stereodivergent strategy for the synthesis of acyclic polysubstituted 1,4-dicarbonyls **19** or **20** (Scheme 6) [45]:

The catalytic aldol reaction of silyl enol ethers is a universal method for C-C bond formation. Gati et al. [46] proposed the triflimide catalyzed *syn*-stereoselective aldol reaction for the synthesis of α,β-dioxyaldehydes **23** and 1,2,3-triols **24** from (*Z*)-tris(trimethylsilyl)silyl enol ethers **21** and aldehydes **22** (Scheme 7):

Iodobenzene acts as a co-catalyst that stabilizes the silylenium cation formed in situ, because the additive seemed to be playing a critical role in affecting the rate of the reaction.

**Scheme 8:**
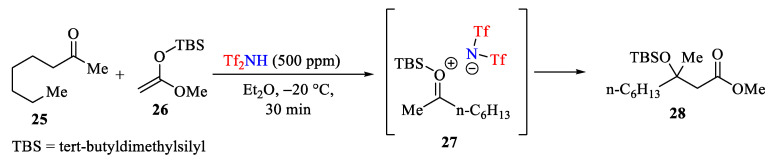
Mukaiyama aldol reaction in the presence of Tf_2_NH.

The Mukaiyama aldol reaction in the presence of “supersilyl” tris(trimethylsilyl)silyl enol ethers **26** with ketone **25** leads diastereoselectively to α,β-dioxyaldehydes, whereas the same reaction in the presence of Tf_2_NH **1** (the reaction of silylketene acetals with ketones) leads to siloxycarbonyl compound **28** [47] (Scheme 8):

This efficient methodology allows for a rapid and stereoselective construction of mono-, bis- and tris-hydroxyaldehydes by mono-, double- and triple-cross-aldol processes, respectively, to produce polyketide-like scaffolds that are particularly useful for the construction of complex natural polyketides [47].

**Scheme 9:**
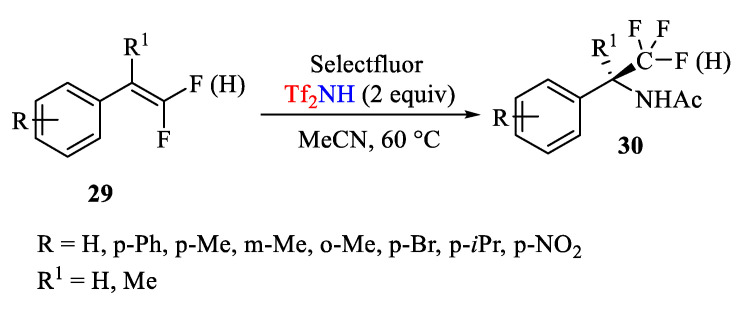
Peterson olefination and aminofluorination of gem-difluoroalkenes and monofluoroalkenes in the presence of Tf_2_NH.

The Peterson olefination in the presence of triflimide **1** [48], as well as the synthesis of α-CF_3_ and α-CF_2_H amines **30** by the aminofluorination of gem-difluoroalkenes and monofluoroalkenes **29**, respectively, was described (Scheme 9) [49]. Selectfluor was used as a source of electrophilic fluorine and acetonitrile as a source of nitrogen.

Triflimide **1** is used as a catalyst in the isoprenylation of aliphatic aldehydes via the (3,3)-sigmatropic rearrangement of *N-*Boc-*N-*(1,1-dimethylallyl)hydrazones [50], in the synthesis of amides from vinyl azides and alcohols [51] and in the rearrangement of *N*-(1-trimethylsilyl)allylhydrazones with the formation of the corresponding vinylsilanes and cyclopropanes [52]. In addition, triflimide activates the silylium catalyst in the reactions of the selective functionalization of azines with the formation of *N-*silylated dihydropyridines [53]. A number of works can be mentioned in which Tf_2_NH was successfully used as a catalyst. These include the synthesis of poly-l-lactide in CO_2_ under plasticization conditions [54], the nucleophilic C-arylation of halopurines leading to N7-substituted purine biaryls [55], reactions of the diastereoselective intramolecular hydride shift in the presence of alkenes [56], the preparation of amides from vinyl azides and alcohols [50], the synthesis of polysubstituted naphthalenes by the reaction of arylacetaldehydes with alkynes (benzenylation reaction) [57,58], glycosylation reactions [59], three-component regioselective synthesis of tetrahydrofuro[2,3-d]oxazoles [60], etc.

## 3. Triflimide Derivatives in Organic Synthesis

### 3.1. AgNTf_2_

**Scheme 10:**
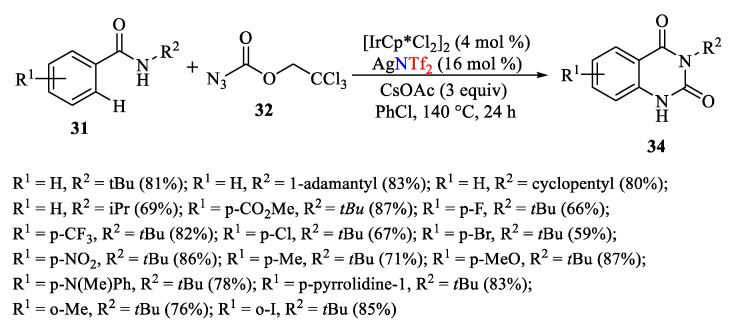
Cyclization of *ortho*- and *para*-substituted benzamides **31** with trichloroethoxycarbonyl azide **32** in the presence of a complex based on silver triflimide **33**.

The direct C–H amidation of substituted benzamides **31** with trichloroethoxycarbonyl azide (TrocN_3_) **32** was reported in the presence of AgNTf_2_ **33** [61]. When cesium acetate is added, the reactions proceed efficiently and with high regioselectivity, affording various functionalized quinazoline-2,4(*1H*,*3H*)-diones **34**, which are important building blocks and key synthetic intermediates from a biological and medical point of view. During the reactions, two new C-N bonds are formed by the successive rupture of the C-H and N-H bonds (Scheme 10)

*N*-Alkylated benzamides **31** with electron-withdrawing or -donating groups in the *para*-position of the phenyl ring cyclize to the corresponding products in good yields. In the same way proceeds the regioselective C-H amidation of *meta*-substituted benzamides **31**. However, substituents in the *meta* position affect both the steric hindrances and electron density of the reaction center of the substrate [61]. The method can be successfully applied to a wide range of oximes, aryl- and alkylsulfonamides as a source of nitrogen [62]. Catalysis by chiral cyclopentadienyl complexes of iridium in combination with AgNTf_2_ **33** is used for CH-arylation of tetralone derivatives with arylboronic esters [63].

The diastereoselective synthesis of spirocyclic pyrrole-2-one-dienone systems was conducted in the presence of AgNTf_2_ **33** in combination with a gold-based complex [64]. A similar procedure was used for the synthesis of diarylmethine-substituted enones [65]. The combination of AgNTf_2_ **33** and the AuCl-based ligand was also successfully used in the cycloisomerization of 1,6-enyne to 3-azabicyclo[4.1.0]heptene [66], as well as in the asymmetric synthesis of fused bicyclic *N*,*O*- and *O*,*O*-acetals [67]. The (C_6_F_5_)_3_PAuCl/AgNTf_2_ catalyzed cyclization of *N-*tosyl-protected 5-benzyl-6-((trimethylsilyl)ethynyl)-1,2,3,4-tetrahydropyridines obtained by the Sonogashira reaction in the presence of enol triflate lactam derivatives gives tetrahydrobenzo[*g*]quinolines, the skeletal framework of which is a recurring motif in natural products [68].

In 2019, an efficient and stereoselective method was developed for the synthesis of (Z)-β-halogenated enamides by the Ritter type Au-catalyzed reaction in the presence of BrettPhosAuCl and AgNTf_2_ **33** using haloalkynes as substrates [69]. The regioselectivity of the reaction was controlled by bulky substituents in the substrates. Other combinations of AgNTf_2_ with Au^+^-based complexes also showed the efficiency in various processes, for example, in the [4 + 3]-annelation of anthranils with 1,5-enynes to form tetrahydrobenzoazepine derivatives [70,71]. The chemoselectivity of the reaction depended on the type of the used alkynes. Silver triflimide AgNTf_2_ **33** was used in one of the stages of the synthesis of new fluorinated symmetric and asymmetric imidazolium salts, as well as of their complexes with various metals [72] and in the diastereoselective synthesis of benzo[5,6]oxepino[2,3-c]pyrroles via the [5 + 2]-annulation of the donor–acceptor type of arylvinyldiazosuccinimide with ketones [73]. The iodination reaction of a wide range of arenes (derivatives of anisole, aniline, acetanilide and phenol) in the presence of AgNTf_2_ **33** and *N-*iodosuccinimide is known [74].

### 3.2. Gold Triflimide

**Scheme 11:**
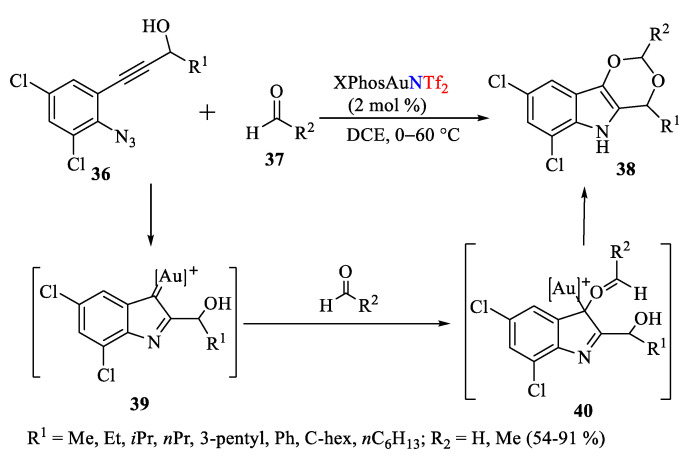
Preparation of [1,3]dioxino[5,4-b]indole derivatives.

Gold triflimides **35** are widely used in organic synthesis. Gold α-iminocarbene complexes have demonstrated good catalytic efficiency in the synthesis of *N-*heterocycles in the last few years [75]. [1,3]Dioxino[5,4-b]indoles **38** have been synthesized by the [4 + 2]-cycloaddition of the 3-indolylidene-Au-carbenium intermediate **39** to aldehydes **37** (Scheme 11). The reaction presumably occurs due to the presence of a hydroxyl group in the starting 3-(2-azidophenyl)prop-2-yn-1-ols **36**. On the other hand, the presence of electron-withdrawing groups such as 3,5-dichloro or 3-cyano in the phenyl ring facilitates the reaction by excluding the competitive attack by the gold-activated alkyne:

A number of syntheses of the oxazino- [76] and pyrazinoindoles [77], pyrroloindoles [78], carbazoles condensed with benzofuran, 1,3H-dibenzo[*a,h*]carbazoles [79], pyridine derivatives [80], pyrroles [81,82,83,84,85], azepines [86] and other polycyclic compounds [87,88,89,90,91,92,93] are known in the literature. In contrast to intramolecular reactions, less is known about the intermolecular formation of gold carbene α-imino intermediates. Nevertheless, these reactions were applied to the synthesis of α,β-unsaturated amidines [94] or 2-aminopyrroles [95] using highly polarized alkynes, for example, ynamides.

**Scheme 12:**
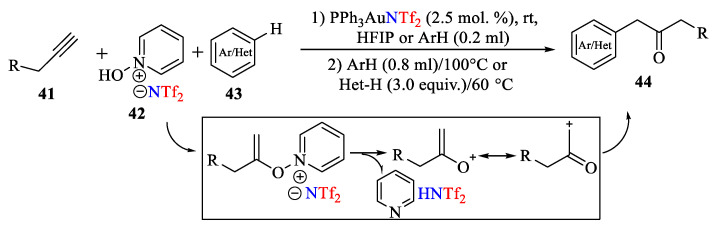
Regioselective functionalization of arenes and heteroarenes to α-aryl- and α-heteroaryl ketones.

In the presence of Ph_3_PAuNTf_2_ 35, the regioselective functionalization of arenes and heteroarenes (derivatives of benzenes, phenols, ethers, indoles, pyrroles, furans and thiophenes) **43** with acetylenes **41** was performed using *N-*alkenoxypyridinium salt **42** as electrophilic alkylating agents for the synthesis of α-aryl- and heteroaryl ketones **44** (Scheme 12) [96]:

N−O bond cleavage might occur prior to the attack by the arene. After N−O bond cleavage, an electrophilic carbocation species is likely produced [96].

Using Ph_3_PAuNTf_2_ **35** makes it possible to synthesize the derivatives of 2-substituted 4-oxo-4-arylbutanal from alkynes and vinyl sulfoxide; five-, six- and seven-membered cycloalkyl-1-ones, for example, the derivatives of tetrahydrocycloalkyl[b]pyrrole, which are pharmaceutical structural units, were easily obtained from 1-cycloalkenyl sulfoxides [97]. The use of Ph_3_PAuNTf_2_ **35** allowed it to synthesize cyclohept[*b*]pyrroles from diynes and pyrroles [98] and to perform glycosylation reactions [99,100]. The combination of Ph_3_PAuNTf_2_ **35** with *N-*iodosuccinimide made it possible to develop a highly efficient synthesis of arenes labeled with radioactive iodine. The reaction represents the first example of the use of homogeneous catalysis in selective synthesis using radioactive materials. The method was used to obtain *meta*-[^125^I]iodobenzylguanidine, a radiopharmaceutical used for imaging and tumor therapy [101].

**Scheme 13:**
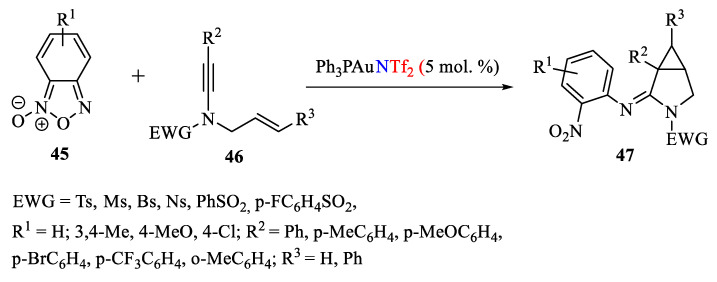
Synthesis of 3 azabicyclo[3.1.0]hexanes from benzofuroxans and *N-*allylynamides catalyzed by Au^+^.

The synthesis of unsymmetrical esters using benzyl, *t*-butyl alcohols as alkylating reagents, also catalyzed by Ph_3_PAuNTf_2_ **35**, was described [102]. An interesting type of reactions proceeding in the presence of gold triflimides complexes are the so-called NT-reactions (*Nitrene Transfer Reaction*). Among various NTRs, in a vast majority of studies, the azides, azirines, isoxazoles, anthranils, pyridinium azaylides and sulfilimines were used as the reagents. These reactions exhibit different activity and selectivity, depending on the chemical nature of the reactants. However, some of the NTRs have significant disadvantages. For example, azides are potentially explosive, and the ylides are poorly reactive in the gold-catalyzed reactions and are not easily available. Shcherbakov et al. [103] have recently demonstrated the gold catalyzed nitrene transfer reaction from benzofuroxans **45** to *N-*allylynamides **46**, resulting in the formation of various 3-azabicyclo[3.1.0]hexanes **47** (Scheme 13):

This highly selective annulation occurs under mild conditions (5 mol % Ph_3_PAuNTf_2_, PhCl, 60 °C) and is applicable to substrates with various functional groups (21 examples, yield ≤ 96%) [103].

### 3.3. LiNTf_2_, Ca(NTf_2_)_2_ and Fe(NTf_2_)_3_

In the presence of LiNTf_2_ **48**, various oxetanes are efficiently opened by C-nucleophiles; for example, silylketene acetals under these conditions give a spectrum of saturated 1,5-oxygen-containing molecules that are part of many natural compounds (for example, polyketides) [104]. It should be noted that when LiNTf_2_ is replaced by TBSNTf_2_ (TBS = = *tert*-butyldimethylsilyl) as a catalyst, the reaction leads to the formation of 1,3,7-oxygen-containing products [104].

**Scheme 14:**
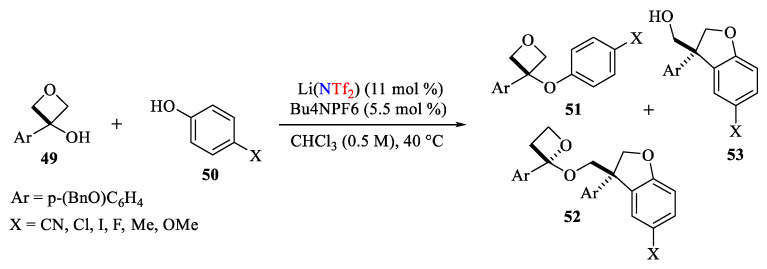
Friedel–Crafts reaction of oxetanols and phenols in the presence of LiNTf_2_.

The formation of oxetane ethers **51**–**52** under the conditions of the Friedel–Crafts reaction between oxetanols **49** and phenols **50** in the presence of LiNTf_2_ was studied in detail by Bull et al. [105]. The introduction of *para*-substituents in the phenyl ring shows a direct dependence of the reaction course and the structure of the products on the nature of the nucleophile, namely, the formation of the kinetic products of O-alkylation versus the thermodynamic products of C-alkylation **53** (Scheme 14) [105]:

For electron-deficient 4-cyanophenol, the only observed product was the product of O-alkylation, while for X = halogen and electron-releasing substituents, after 1 h, a mixture of C- and O-alkylated products was formed, and after 20 h, only the C-alkylation products were observed in the mixture. In addition, a number of oxetane ester derivatives were isolated, which are new potential bioisosteres for esters of carboxylic acids [105]. When the reaction was monitored using ^1^H NMR spectroscopy without a nucleophile present, a rapid degradation of the starting material occurred. A small amount of an aldehyde side product was identified; however, the majority of the material was unaccounted for. An insoluble precipitate was observed, indicating the possible formation of a polymeric species under the reaction conditions resulting from ring opening of the oxetane, promoted by the formation of the oxetane carbocation in the absence of a better nucleophile than the substrate itself [105].

**Scheme 15:**
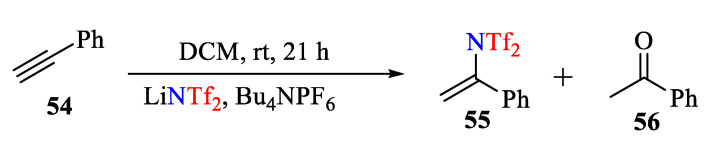
Hydroaminosulfonation of phenylacetylene.

The chemo-, regio- and stereoselective addition of triflimide (from LiNTf_2_ **48**) to alkynes **54** is known. The reaction represents the second sample of the rare class of vinyl triflimides **55** in good yields (Scheme 15) [106]:

*N*-vinyl triflimides were first described in the work [107]. 

**Scheme 16:**
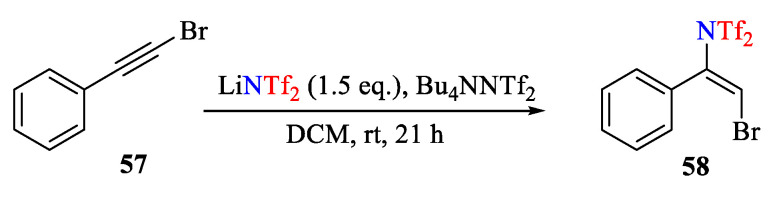
Synthesis of bromvinyl triflimide.

The reaction was performed by adding phenylacetylene to the dichloromethane solution of 1.5 eq. LiNTf_2_ **48** and Bu_4_NNTf_2_ to increase the solubility of LiNTf_2_ **48**. The regioselectivity of the addition is determined by the cation-stabilizing effect of the α-aryl substituent [108]: (Scheme 16).

**Scheme 17:**
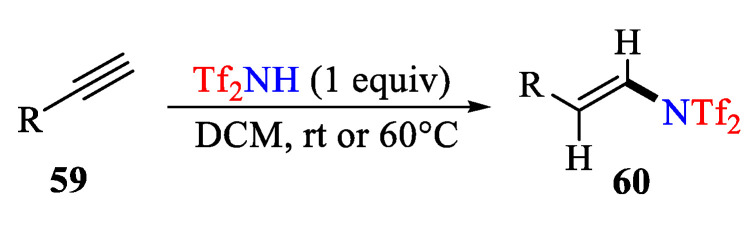
Reactions of aryl-, vinyl-, alkyl- and silyl-substituted terminal alkynes with triflimide.

**Scheme 18:**
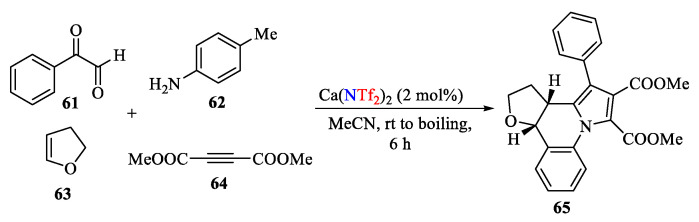
Calcium triflimide-catalyzed stereoselective tandem of [4 + 2]- and [3 + 2]-annulation for the synthesis of ropyrrolo[1,2a]quinolines.

A similar reaction of Si-substituted alkynes **59** and Tf_2_NH **1** with the formation of vinyl triflimides **60** is known. The process proceeds in dichloromethane with slight heating and does not require the presence of additional catalysts (Scheme 17) [109]:

In the presence of calcium triflimide **66**, the annulation reaction of an aldehyde **61**, amine **62**, alkene **63** and alkyne **64** takes place. The process proceeds with the formation of fused pyrrolo[1,2a]quinolones 65 with exclusive syn-diastereoselectivity and good yields (Scheme 18):

This tandem annulation process delivers products that generally comprise four or five rings fused in an angular fashion [110].

For example, in the reactions of furylcarbinol with amines under the action of Ca(NTf_2_)_2_, the Aza-Piancatelli rearrangement occurs, and after the addition of Et_3_N to the reaction mixture, an intramolecular Michael addition proceeds with the formation of the corresponding tetrahydrobenzo[b]azepines in 33–93% yields [111]. The reactions of N,O-acetals with vinylboronic acids in the presence of Ca(NTf_2_)_2_ lead to the formation of isoindolinones in good to quantitative yields [112].

**Scheme 19:**
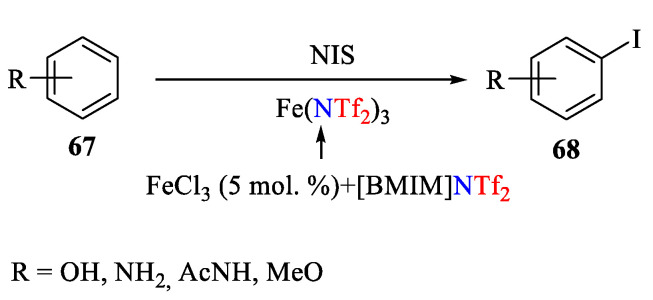
Fe(NTf_2_)_3_-catalyzed iodination of arenes.

Iron(III) triflimide salt **67** (formed in situ from FeCl_3_ and AgNTf_2_) is an efficient catalyst for addition reactions [113]. An alternative method for preparing Fe(NTf_2_)_3_ that avoids the use of additional metal salts consists of dissolving FeCl_3_ in a readily available and inexpensive ionic liquid [BMIM]-NTf_2_ (1-butyl-3-methylimidazolium bis(trifluoromethanesulfonyl)imide). The combination of FeCl_3_ with the ionic liquid resistant to air oxygen accelerates the reaction, and the FeCl_3_+[BMIM]NTf_2_ system has proven to be ideal for the direct iodination of aromatic compounds **67** in the presence of *N-*iodosuccinimide (NIS) with good yields (58–91%) [114] (Scheme 19):

The reaction with *N-*chlorosuccinimide (NCS) proceeds similarly [100,115]. The yields of the corresponding chlorinated arenes (26 examples) ranged from 53 to 97%. The reaction was used for the mono- and di-chlorination of a number of target products, such as nitrofungin, the anti-bacterial agent chloroxylenol and the herbicide chloroxynil [115].

**Scheme 20:**
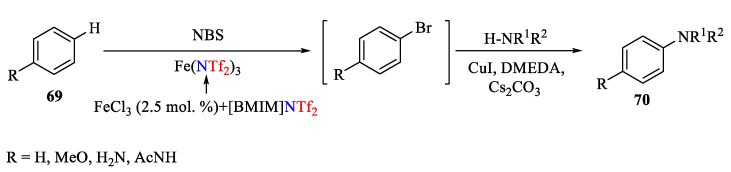
Strategy for regioselective CH-amination of arenes in the presence of Fe(NTf_2_)_3_/CuI.

In the presence of Fe(III) and Cu(I), a regioselective reaction of the *para*-amination of the activated arenes **69** occurs via bromination with *N-*bromosuccinimide (NBS) (yields 51–95%) [116] (Scheme 20):

The reaction involves the bromination of an aryl substrate in the presence of Fe(NTf_2_)_3_ followed by an *N-*arylation reaction catalyzed by Cu(I). A similar system was used for the synthesis of 2-arylbenzoxazoles and 2-arylbenzothiazoles from *N-*arylbenzamides. [117].

## 4. Synthesis of Biologically Active Triflamide Derivatives

**Scheme 21:**
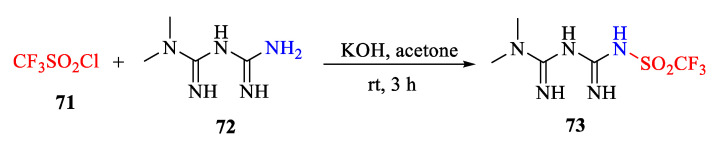
Synthesis of *N-*trifluoromethanesulfonyl derivative of metformin.

Thrombosis is the main pathogenesis that causes the low curability of ischemic stroke and is often the cause of death and disability worldwide. Metformin **72**, a biguanidine derivative, is a drug for the treatment of type 2 diabetes mellitus, which alleviates the course of ischemic stroke in patients with diabetes mellitus. Based on triflamide and metformin, a promising drug for the treatment of stroke was obtained [118] (Scheme 21):

The compound is prepared by the simple treatment of metformin **72** with trifluoromethanesulfonyl chloride **71** in anhydrous acetone in the presence of catalytic amounts of KOH. The target product was obtained in an 85% yield. The compound inhibits the formation of human platelets, including the reduction of platelet aggregation, adhesion and clot retraction, strongly inhibits the formation of blood clots in arteries, reduces the size and compactness of blood clots in stroke, reduces damage to nerve function and mortality and does not cause severe toxicity and tissue damage [118]. As shown later [119], this modified metformin exhibits selective biological activity against breast cancer cells (MFC-7). Together with its anti-thrombotic properties, which is very important in the treatment of cancer, the compound is a promising drug for the therapy of cancer [119,120].

**Scheme 22:**
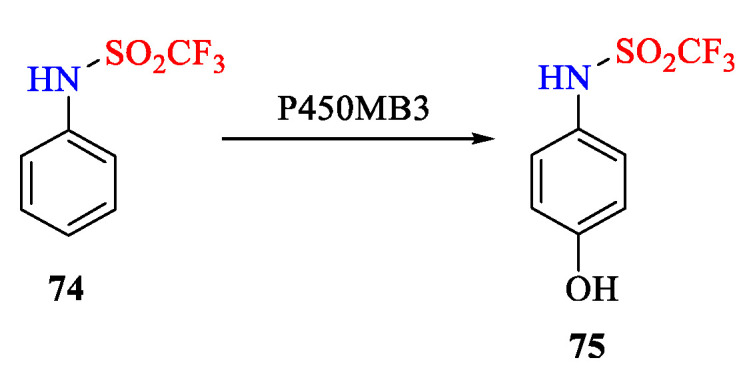
Biocatalytic hydroxylation of *N-*trifluoromethanesulfonyl substituted aniline.

*N-*Trifluoromethanesulfonyl-substituted anilines **75** proved to be effective substrates for biocatalytic hydroxylation with the formation of 4-aminophenols in the presence of a number of cytochromes of the P450BM3 family. The reactions proceeded with 100% conversion. Similar results were shown by *N*-trifluoroacetyl protection at the nitrogen atom of aniline. Ac- or Boc-derivatives of anilines showed only a 17 and 66% conversion, respectively [121] (Scheme 22):

P450-catalysed arene hydroxylation is accepted to occur via an NIH-shift within an iminium intermediate to give a dienone that re-aromatizes to phenol [121]. Biocatalytic hydroxylation reactions are used for the synthesis of drugs, agrochemicals and their metabolites. In the case of *N-*trifluoromethanesulfonyl derivatives, the reaction proved to be effective both in preparative and screening variants [121].

**Scheme 23:**
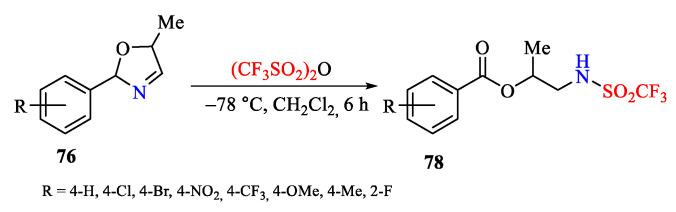
Ring-opening of the 5-methyl-2-phenyl-4,5-dihydrooxazole system by triflic anhydride.

The reaction of 5-methyl-2-phenyl-4,5-dihydrooxazole **76** with trifluoromethanesulfonic acid anhydride **77** leads to the formation of trifluoromethanesulfonamido)prop-2-yl esters of phenyl-substituted derivatives of benzoic acid **78** [122] (Scheme 23):

The product yield varies from 56 to 88%. The reaction does not require the presence of bases. Derivatives with R = 4-Cl, 4-NO_2_ and 4-CF_3_ show high cytotoxicity against six human cancer cell lines (U251 (glioblastoma), PC-3 (prostate adenocarcinoma), K-562 (chronic myelogenous leukemia), HCT-15 (colorectal adenocarcinoma), MCF-7 (breast adenocarcinoma), SKLU-1 (lung adenocarcinoma) and the 4-CF_3_ derivative was active against human gum cancer cells (FGH  (gingival fibroblastoma) [122].

**Scheme 24:**
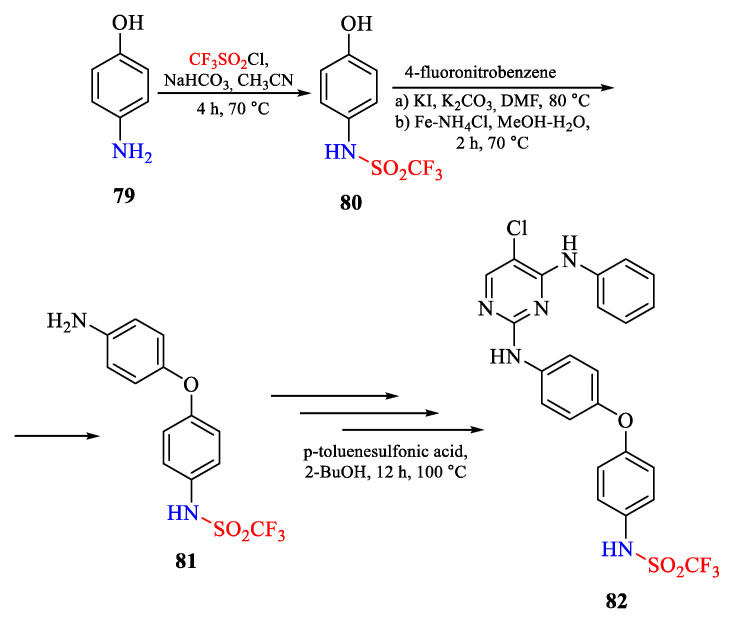
Synthesis of triflamide-containing *anti*-pancreatic carcinoma derivative.

A triflamide derivative of diphenylpyrimidine **82** is known, which exhibits excellent activity against the proliferation of pancreatic carcinoma cells (AsPC-1, Panc-1, BxPC-3), lymphoblastic leukemia cells (Ramos) and some lung cancer cells (Scheme 24):

4-Aminephenol was converted to *N-*phenyl triflamide **80** intermediates under the action of trifluoromethane sulfonic anhydride. Then, **80** were reacted with 4-fluoronitrobenzene to form the intermediate, which were conveniently converted to the amine derivative **81** by using the Fe-NH_4_Cl reduction condition. Additionally, under the action of the p-toluenesulfonic acid reagent, **81** was reacted with the 2-chlorine pyrimidine derivative to generate the title molecule **82** [123].

**Scheme 25:**
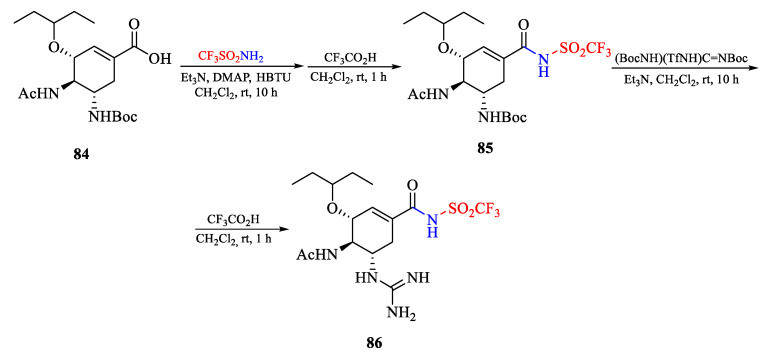
Synthesis of oseltamivir acyl triflamide derivative.

On the basis of triflamide **83**, a derivative of oseltamivir **84**, an anti-viral drug used to treat various types of influenza, was obtained (Scheme 25):

Coupling reactions of acid **84** with triflamide **83** were carried out, followed by the removal of the Boc protecting group with TFA, to afford acyl triflamide **85**. The amino group in **85** was further elaborated to the guanidino group by treatment with 1,3-di-Boc-2-(trifluoromethylsulfonyl)guanidine to afford GOC-sulfonamides **86** after the removal of the Boc groups. The compound 86 exhibited high inhibitory activity against the H1N1 influenza virus, with the final yield of the product being 39% [123,124].

**Scheme 26:**
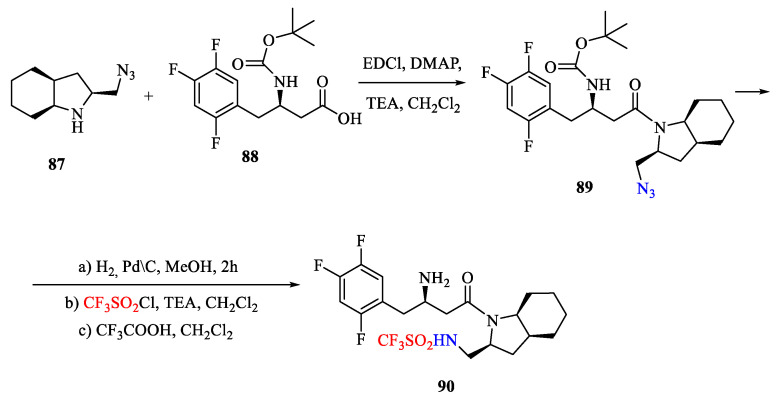
Synthesis of DPP-4 inhibitor.

Type 2 diabetes mellitus is a progressive metabolic disorder characterized by high blood glucose and high endogenous insulin levels. Diabetes mellitus causes serious vascular complications, heart disease, kidney failure and blindness [125]. Glucagon-like peptide-1 (GLP-1) is a potent anti-hyperglycemic hormone that induces the glucose-dependent stimulation of insulin secretion while simultaneously inhibiting glucagon secretion. However, active GLP-1 is rapidly degraded by the dipeptidyl peptidase-4 (DPP-4) enzyme. Therefore, the inhibition of DPP-4 is a new approach to the treatment of type 2 diabetes. Based on triflamide, a DPP-4 inhibitor **90** in the low micromolar range was obtained, the pharmacokinetic profile of which was suitable for clinical use (Scheme 26):

Compound **90** was synthesized according to Scheme 26. The condensation of **87** with **88** provided compound **89**, which was then reduced by H_2_ and condensed with sulfonyl chloride and was followed by the deprotection of Boc by treatment with TFA in CH_2_Cl_2_, which provided target compound **90** [125].

**Scheme 27:**
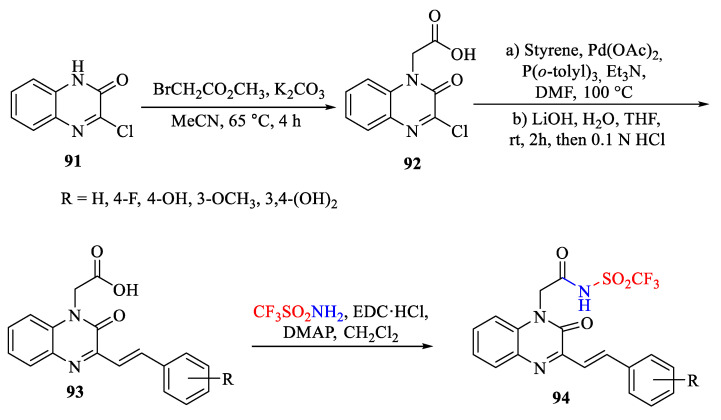
Synthesis of quinoxalinone-based triflamide derivatives.

Triflamide derivatives have been obtained that exhibit anti-diabetic activity as inhibitors of aldose reductase (ALR2), aldehyde reductase (ALR1) and antioxidant activity. These *N-*acyl triflamide derivatives **94** are obtained on the basis of the quinoxalinone **91** framework [126] (Scheme 27):

3-Chloro-quinoxalin-2(*1H*)-one **91** was alkylated with methyl bromoacetate to form methyl ester **92** as a key intermediate. Then, **92** was subjected to the Heck coupling reaction with the corresponding styrenes and then to hydrolysis with lithium hydroxide, and the carboxylic acid **93** was afforded. Finally, **93** was treated with triflamide **83** in the presence of EDC·HCl and DMAP to obtain the desired compound **94**.

The yield of the final reaction products was 57–70%. The product 94 with R = 3,4-(OH)_2_ exhibited the highest inhibitory activity. It also exhibited extraordinary antioxidant activity, which was even higher than that of the commercial drug Trolox (6-hydroxy-2,5,7,8-tetramethylchroman-2-carboxylic acid), a water-soluble analogue of vitamin E [126].

**Scheme 28:**
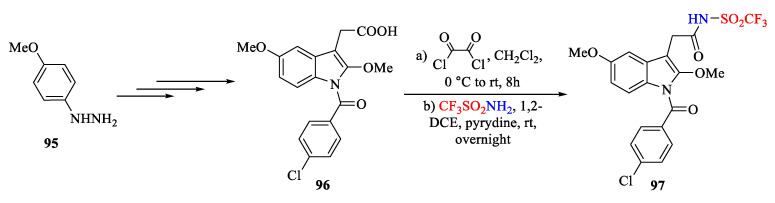
Synthesis of indomethacin triflamide analogues.

The triflamide derivative of indomethacin **97** is known, which is promising in the treatment of prostate cancer, showing high selectivity and good inhibitory properties in the treatment of this disease. The yield of the product was 70% [127] (Scheme 28):

Compound **96** was obtained from 4-chloro-*N-*(4-methoxyphenyl)- benzohydrazide hydrochloride (obtained from **95**) by the refluxing with slight excess of 4-oxobutanoic acid in AcOH, respectively. Then, **96** and triflamide **83** were dissolved in 2 mL of 1,2-dichloro ethane (DCE) under stirring to obtain compound **97**.

**Scheme 29:**
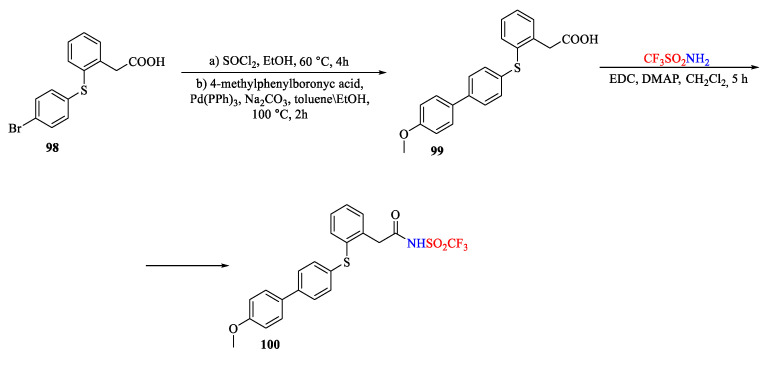
Synthesis of triflamide-containing selective matrix metalloproteinase-12 (MMP-12) inhibitor.

Triflamide has been used to synthesize the selective inhibitor of matrix metalloproteinase-12 (MMP-12) **100**. The inhibition of the reactions of this enzyme often plays an important role in the treatment of lung, inflammatory and cardiovascular diseases [128] (Scheme 29):

Diarylsulfide derivative **100** was prepared as reported in Scheme 29. Compound **98** was protected as an ethyl ester by treatment with thionyl chloride (SOCl_2_) in ethanol. This step was necessary to improve the subsequent cross-coupling reaction, which exhibited a poor yield if conducted on carboxylic acid. A palladium-catalyzed cross-coupling reaction (under Suzuki conditions) of protected aryl bromide **98** with 4-methoxyphenylboronic acid afforded biphenyl derivative **99**. Compound **99** was converted into sulfonamide **100** by condensation with triflamide **83**, respectively, in the presence of *N-*(3-(dimethylamino)propyl)-N′-ethylcarbodiimide (EDC) and 4- dimethylaminopyridine (DMAP) using dichloromethane as a solvent.

The yield of the target product **100** was 80%. A similar sulfone was also obtained, but its selectivity and inhibitory activity were much lower [128].

**Scheme 30:**
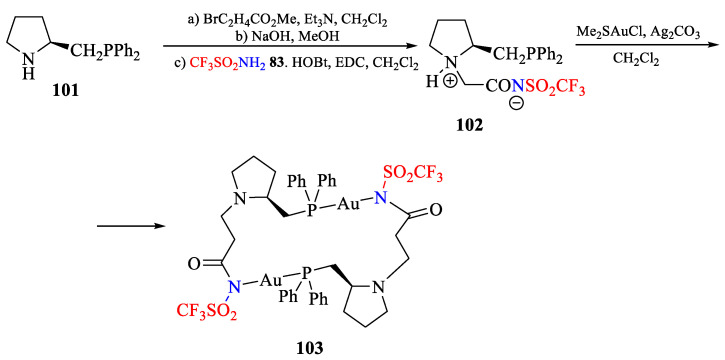
Synthesis of chiral complexes based on *N-*trifluoromethanesulfonyl-substituted amino acids and Au(I).

Chiral complex **103** was obtained on the basis of *N-*trifluoromethanesulfonyl substituted amino acids **102** and Au(I). The complex **103** exhibited in vitro cytotoxicity against breast cancer cells with limited toxicity to healthy epithelial cells [129] (Scheme 30):

Ligand **102,** derived from the polar amino acid proline’ Secondary amine **101,** was alkylated with methyl 3-bromopropanoate16 and the ester was subsequently saponified to the corresponding carboxylic acid. An EDCmediated amidification reaction afforded the corresponding triflic propionamide **102**. Complex **103** was derived from the dimethylsulfide gold chloride complex; the two different structures depend on the initial presence of a silver(I) salt. The unusual dimeric complex 6 was obtained in one synthetic operation by ligand metathesis between a silver amide salt generated in situ from ligand **102**. The complex **103** was obtained in a quantitative yield. These complexes of triflamide and gold(I) derivatives have the ability to selectively accumulate in adenocarcinoma cells, which makes it possible to effectively treat cancer in elderly patients and for slow-growing tumors, that is, just in those cases when traditional cancer therapy aimed at combating rapidly dividing cells cannot be used [129].

**Scheme 31:**
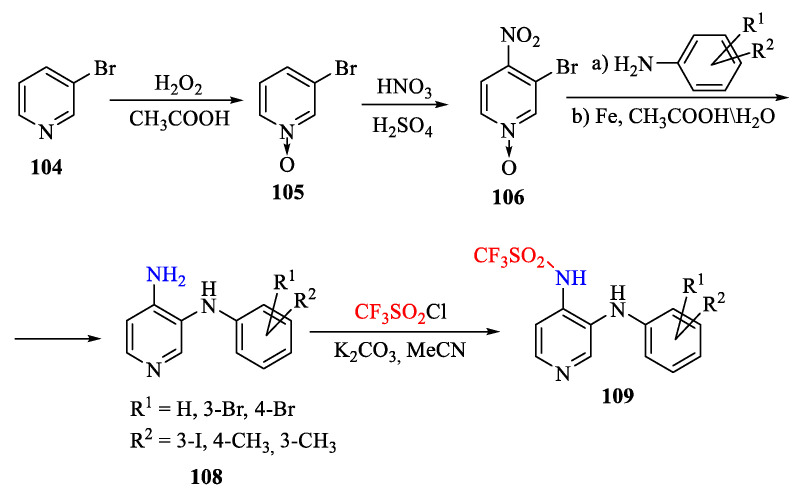
Synthesis of non-steroidal anti-inflammatory drugs-derivatives of triflamide.

Triflamide derivatives are known, on the basis of which non-steroidal anti-inflammatory drugs were obtained, similar in action to nimesulide or celecoxib, which are safe for the gastrointestinal tract and cardiovascular system. The resulting compounds **109** are characterized by a high anti-inflammatory activity, similar to or higher than that of nimesulide or celecoxib, as well as a high COX-2/COX-1 selectivity [130] (Scheme 31):

The synthetic pathway for compound **109** can be summarized as follows: 3-bromopyridine **104** is first oxidized by a mixture of acetic acid and hydrogen peroxide to afford 3-bromopyridine *N-*oxide **105**, which is then nitrated at the 4- position by a nitric and sulfuric acid medium to provide the key intermediate 3-bromo-4-nitropyridine *N-*oxide **106**. The NH-bridge is achieved by the reaction of intermediate **106** with properly substituted anilines **107**. The 4-aminopyridine intermediates **108** are obtained after the simultaneous reduction of the nitro and the pyridine *N*-oxide moieties using iron in an acetic acid and water medium. The pyridine analogs of nimesulide **109** are obtained by the reaction of the aminopyridine intermediates **108** with trifluoromethylsulfonyl chloride in acetonitrile in the presence of potassium carbonate. The products **109** were obtained in a 35–77% yield [130].

**Scheme 32:**
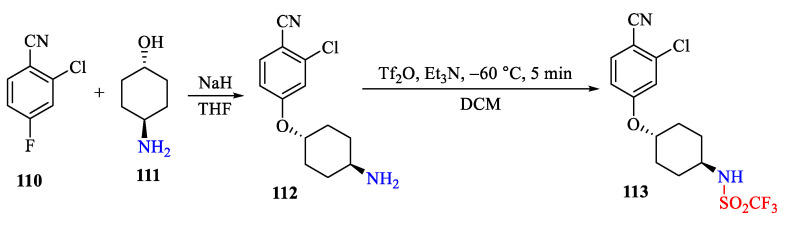
Synthesis of trifluoromethanesulfonyl-based antagonists of the progesterone receptor.

Triflamide derivatives exhibit biological activity as progesterone receptor antagonists. The product **113** presented in Scheme 32 is a potentially effective drug in the treatment of diseases of the female reproductive system, including endometriosis [131] (Scheme 32):

The synthetic route towards compound **113** is described in Scheme 32. The deprotonation of cyclohexanol **111** with NaH in THF followed by the addition of fluorobenzonitrile **110** furnished cyclohexylamine **112** in a good yield. Compound **113** was synthesized by the triflation of cyclohexylamine 112 using triflic anhydride and triethylamine in DCM at −60 °C. The yield of the final product **113** was 92%. The product **113** also inhibited drug metabolism by cytochromes P450, CYP 2C [131].

**Scheme 33:**
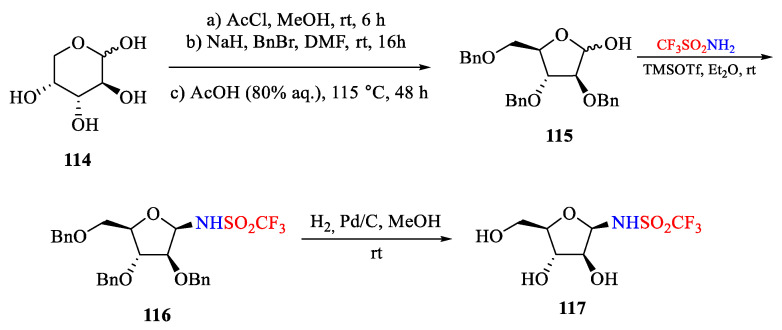
Synthesis of *N-*glycosyl triflamide.

*N-*Trifluoromethanesulfonyl-substituted derivatives **117** were obtained, exhibiting anti-mycobacterial activity, and could potentially become new anti-microbial drugs, including for the treatment of tuberculosis [132] (Scheme 33):

The synthetic route towards compound **117** is presented in Scheme 33. Acetyl chloride was added to a solution of **114** in methanol under nitrogen. The reaction was stirred for 3 h at room temperature. Sodium hydride and benzyl bromide were added. The reaction was cooled in an ice bath, quenched by the addition of methanol and then concentrated in vacuo. Then, the residue was dissolved in a mixture of water and acetic acid to afford hemiacetal **115**. Compound **115** and triflamide **83** were stirred at room temperature in dry diethyl ether in the presence TMSOTf to give compound **116**. Compound **117** was obtained by reduction with 10% activated Pd/C. The *N-*furanosyl triflamide 117 was obtained in a 44% yield [132]. Compound **117** was configurationally stable in aqueous solutions, in contrast to its other analogues obtained from alkylsulfonamides. *N-*Acyltriflamides also exhibited anti-mycobacterial activity [133].

**Scheme 34:**
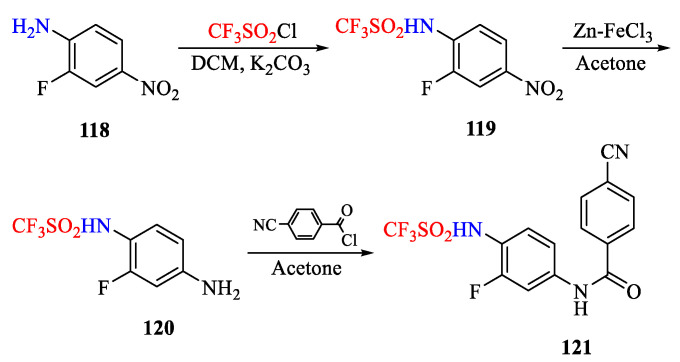
Synthesis of trifluoromethanesulfonyl-based tubulin inhibitor.

Triflamide derivatives exhibit biological activity against the causative agent of sleeping sickness (African trypanosomiasis) [134]. Currently, there are five drugs for the treatment of this disease, including suramin, pentamidine, melarsoprol, eflornithine and nifurtimox [135]. However, their use is accompanied by a number of serious side effects and difficulties: (1) high toxicity; (2) the necessity to be injected intramuscularly or intravenously, which creates difficulties in an epidemic area with limited medical resources; (3) a narrow antitrypanosomous spectrum of action; and (4) high cost. In general, these drugs are not effective in treating the disease, and there is an urgent need to develop more effective and inexpensive chemotherapeutic agents for the treatment of trypanosomiasis. The treatment of this disease involves blocking the processes of polymerization/depolymerization of the tubulin protein, which is necessary for the division of pathogen cells and their movement. On the basis of the triflamide derivative, a selective tubulin inhibitor 121 was obtained, which can be used to treat sleeping sickness [134] (Scheme 34):

Triflamide derivative **119** was prepared from aryl-substituted 2-amino-5-nitrophenol **118** by adding it with trifluoromethanesulfonic chloride in anhydrous DCM and K_2_CO_3_. Compound **119** was dissolved in acetone; then, Zn and FeCl_3_ were added into the solution. When the reaction completed, the corresponding benzoyl chloride was added and product **121** was collected by filtration and purified by recrystallization in ethanol/water. The yield of the final product **121** of the reaction was 47% [134].

**Scheme 35:**
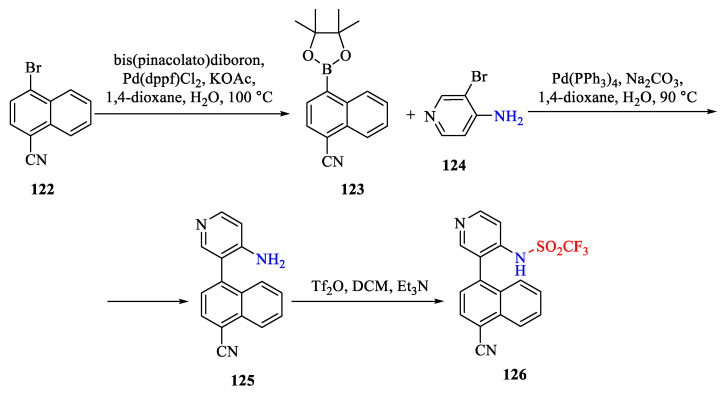
Synthesis of triflamide-containing hURAT1 inhibitor.

The derivative of triflamide **126** is known to exhibit biological activity and act as an inhibitor of the transporter derivatives of uric acid (hURAT1). In medicine, there are only three options for drugs of this kind, although they are extremely necessary in the treatment of hyperuricemia, which subsequently causes many diseases, such as gout, arterial hypertension, chronic kidney disease and some cardiovascular diseases [136] (Scheme 35):

Triflamide compound **126** was synthesized by following Scheme 35. Bis(pinacolato)diboron reacted with **122** under the catalyst of Pd(dppf)Cl_2_, which gave compound **123**. Further Suzuki coupling of **123** with 4-amino-3-bromopyridine **124** provided **125**, which subsequently reacted with CF_3_SO_2_Cl under the conditions of Et_3_N in DCM to afford compound **126** [136].

**Scheme 36:**
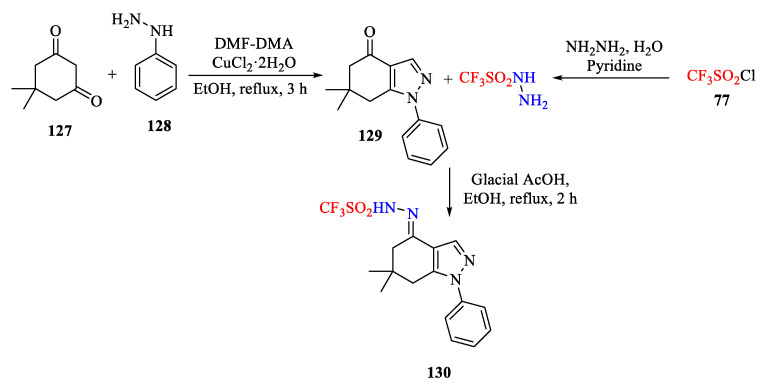
Synthesis of triflamide-containing α-amylase enzyme inhibitor.

Based on triflamide, a promising inhibitor of the α-amylase enzyme **130** was obtained [137] (Scheme 36):

Sulfonohydrazide-substituted indazole **130** was synthesized by a multi-step reaction. In the first step, 4-oxyindazole **129** was formed by reacting dimedone **127**, dimethylformamide dimethylacetal (DMF-DMA) and phenylhydrazine **128** in the presence of a catalytic amount of CuCl_2_ in ethanol for 3 h. In the next step, 4-oxyindazole **129** was treated with a sulfonylhydrazide derivative in ethanol in the presence of pyridine as a catalyst and was refluxed for 2 h to obtain the desired sulfonohydrazide-substituted indazole **130** (Scheme 36). Reducing the activity of the α-amylase enzyme is one of the best treatments for type 2 diabetes. For example, acarbose, voglibose and miglitol are commercially available α-amylase enzyme inhibitors used to treat type II diabetes mellitus. However, these agents have some side effects such as flatulence, diarrhea and abdominal discomfort, so other anti-diabetic agents are always recommended for greater effectiveness [137].

**Scheme 37:**
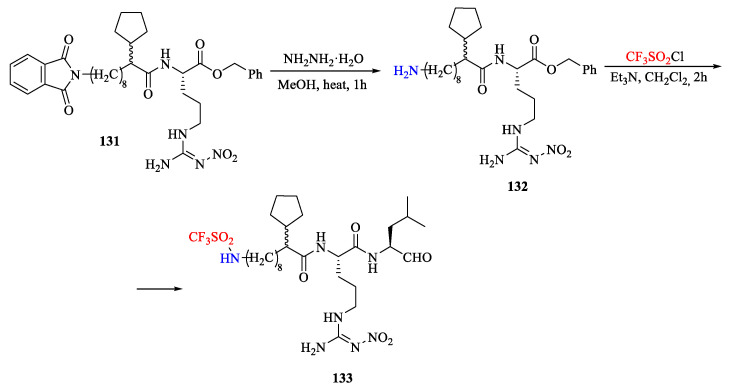
Synthesis of triflamide-containing peptidomimetic.

Triflamide derivatives are used for the synthesis of inhibitors of biochemical processes by introducing peptidomimetics into biochemical reactions. A triflamide-containing component of the proteasome complex **133** was obtained, exhibiting activity similar to chymotrypsin. Similar compounds are used for cancer therapy. The product **133** yield was 80% [138] (Scheme 37):

The synthesis of the target compound is presented in Scheme 37. The treatment of compound **131** with hydrazine generated compound **132,** which was treated with trifluoromethylsulfonyl chloride in a basic media to generate sulfonamide **133** over two steps [138].

**Scheme 38:**
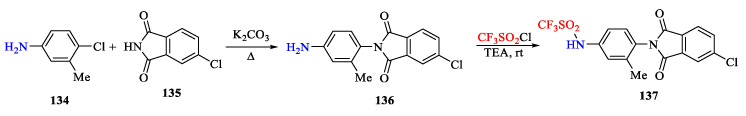
Synthesis of triflamide-containing anti-retroviral agent.

Scheme 38 shows a simple synthesis of a compound **137** exhibiting high anti-retroviral activity. The compound showed good activity against HIV at nanomolar concentrations, being an effective inhibitor of HIV-1 replication [139] (Scheme 38):

The synthesis of triflamide derivative **137** was achieved from the key intermediate, *N-*(4-amino-2-methylphenyl)-4-chloro-phthalimine **136**, which was synthesized from 4-chlorophthalimine **135** and 4-chloro-3- methylaniline **134**. Further, **136**, when treated with appropriate CF_3_SO_2_Cl in the presence of TEA and DCM at room temperature, gave the respective sulfonamide **137**.

The resulting isoindolindione **137** gave a good performance in overcoming the hematoencephalic barrier. Obtaining such drugs is important because there is a demand in medicine for drugs for highly active anti-retroviral therapy, which is necessary to curb the progression of HIV disease and increase the survival of HIV-infected patients, as well as due to the high resistance of retroviruses [139].

## 5. Triflamide Derivatives in Organic Synthesis

**Scheme 39:**
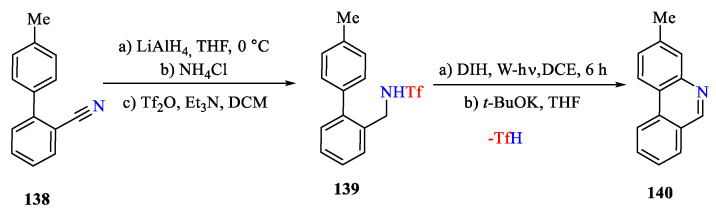
Preparation of phenanthridine from *N-*(*o*-arylbenzyl) trifluoromethanesulfonamides with 1,3-diiodo-5,5-dimethylhydantoin.

Arylbenzyltrifluoromethanesulfonamides **139** are starting compounds for the synthesis of phenanthridine derivatives **140**, which are among the most important basic nitrogen-containing heteroaromatic structures. Phenanthridine fragments are part of natural compounds and have powerful anti-bacterial and anti-tumor activity [140]. O-Arylbenzyltrifluoromethanesulfonamides **139** are obtained by the reduction of the corresponding CN-diaryls **138** followed by treatment with triflic anhydride [140] (Scheme 39):

Then, the product undergoes intramolecular oxidative cyclization at the aromatic ring in the presence of DIH in 1,2-dichloroethane under irradiation with a tungsten lamp with the formation of 5-trifluoromethanesulfonyl-5,6-dihydrophenanthridine, which, by the action of t-BuOK in THF, gives the target phenanthridine **140** (79%) [140].

**Scheme 40:**
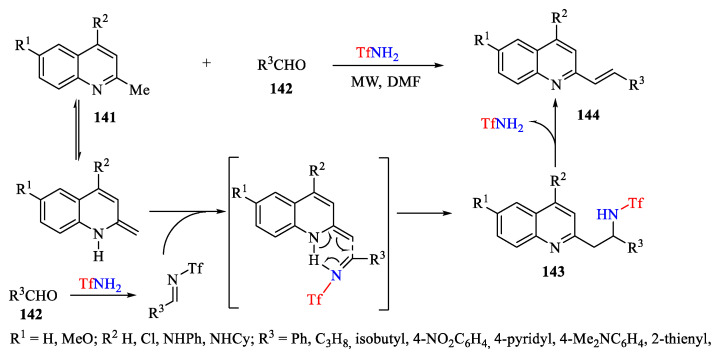
Microwave-assisted synthesis of 2-vinylquinolines in the presence of TfNH_2_.

An olefination reaction of 2-methylquinoline in the presence of triflamide was shown to proceed in the presence of aldehydes under the action of microwave radiation with the formation of 2-vinylquinolines **144** [141] (Scheme 40):

Not only derivatives of aliphatic aldehydes or benzaldehydes **142**, but also heteroaromatic aldehydes (pyridinecarboxaldehyde and thiophenecarboxaldehyde) can be involved in the reaction. Triflamide **83** is added in the excess of 20% with respect to the aldehyde. As seen in Scheme 40, the microwave irradiation of TfNH_2_ **83** gives the corresponding aldimine in situ. The corresponding alkene **144** is formed by the elimination of the triflamide molecule from **143**. Some of the obtained alkenes have high anti-malarial biological activity [141].

Various triflamide compounds are widely used as organyl catalysts that show high stereoselectivity and asymmetric transformations [142,143,144,145,146,147,148,149].

**Scheme 41:**
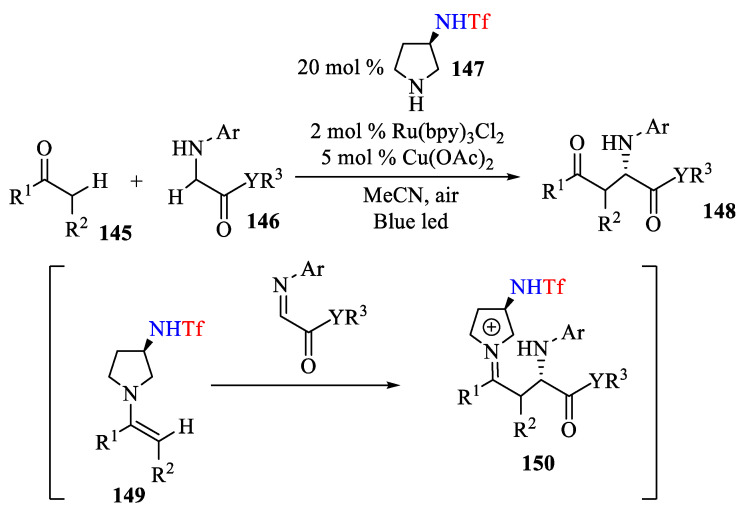
Asymmetric Mannich-type reaction for the C−H Alkylation in the presence of TfNH-containing organyl catalyst.

Triflamide derivatives **147** are used in the enantioselective cross-coupling of glycine **146** derivatives with ketones and aldehydes **145** [143] (Scheme 41):

The photoinduced process includes the oxidation of glycine derivatives to an imine intermediate, which enters the asymmetric Mannich reaction with an enamine **149** intermediate adduct formed in situ from a ketone or aldehyde **145** and a chiral TfNH-containing organic catalyst **147**. The method allows one to create a new C–C bond with the formation of new stereocenters without the additional functionalization of the substrates [143].

Similar to organic catalysts, 2-(trifluoromethanesulfonamidoalkyl)pyrrolidines and their D-prolinamides were used in the addition of aldehydes to β-nitroalkenes at room temperature. The reaction in this case proceeded without additional reagents and catalysts and led to the formation of γ-nitroaldehydes in a quantitative yield and high enantio- and diastereoselectivity [144].

**Scheme 42:**
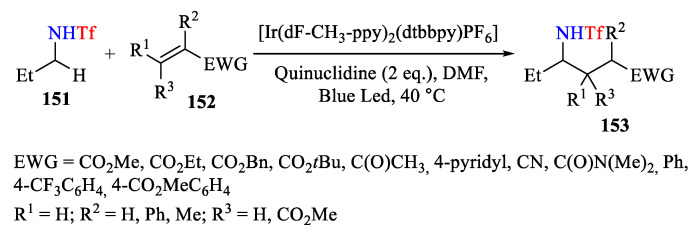
C-H Alkylation of *N-*propyl substituted Triflamide.

*N-*Propyltriflamide **151** reacts with activated alkenes **152** in the presence of an Ir(III) complex as a photocatalyst and a base (quinuclidine) [150] (Scheme 42):

The more stable nitrogen-centered radical present on triflamide acts as an intermolecular hydrogen-atom abstractor to the deprotonated triflamides in the solution, which possess highly activated α-CH bonds due to their anionic character. This pivot in reactivity allows for the selective functionalization of both α- and δ-CH bonds depending on the installed nitrogen protecting group. The presence of a trifluoromethanesulfonyl group at the nitrogen atom ensures the complete deprotonation of the NH bond and makes the α-C-H bond more “hydride” and susceptible to hydrogen-atom transfer (HAT) under the action of quinuclidine and a photocatalyst. The reaction allows for the formation of a new C-C bond due to the combination of α-amino radicals and electron-deficient alkenes. The yields of **153** vary from 32 to 63% [150].

**Scheme 43:**
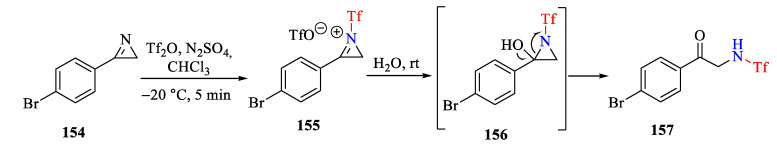
Formation of *N-*Triflyl acetophenone derivative.

The reaction of 2*H*-azirine **154** with a slight excess of Tf_2_O in chloroform leads to the formation of the *N-*acetophenone derivative of triflamide **157** [151] (Scheme 43):

2*H*-Azirine **154** is activated by a nucleophilic attack by the lone nitrogen pair on the highly electrophilic Tf_2_O, resulting in the formation of the triflate of the *N-*triflylazirinium cation **155**. In the presence of water in the reaction medium, the iminium ion is hydrolyzed to N-triflyl α-aminoacetophenone **157** (71%). In anhydrous medium, when 2-chloropyridines are added, this *2H*-azirine reacts in the presence of Tf_2_O to give imidazo[1,2*-a*]pyridines [151].

**Scheme 44:**
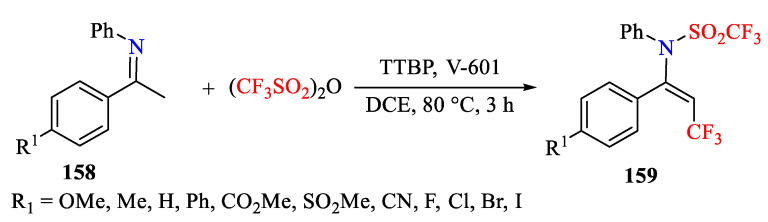
One-pot synthesis of CF_3_-substituted vinyl trifluoromethanesulfonamides from imines and triflic anhydride.

A unique one-pot reaction of the radical trifluoromethanesulfonation and trifluoromethylation of imines **158** is known, which proceeds with the formation of CF_3_-substituted *N-*vinyl triflamides **159** [152] (Scheme 44):

The reaction starts with the formation of *N-*vinyltriflamides, which are the sources of the CF_3_ group. In this case, the *N-*vinylatriflamides obtained at the first stage of the reaction further react as bifunctional reagents, acting as both sources of trifluoromethyl radicals and acceptors of these radicals. The yields vary from 34 to 61% [152].

**Scheme 45:**
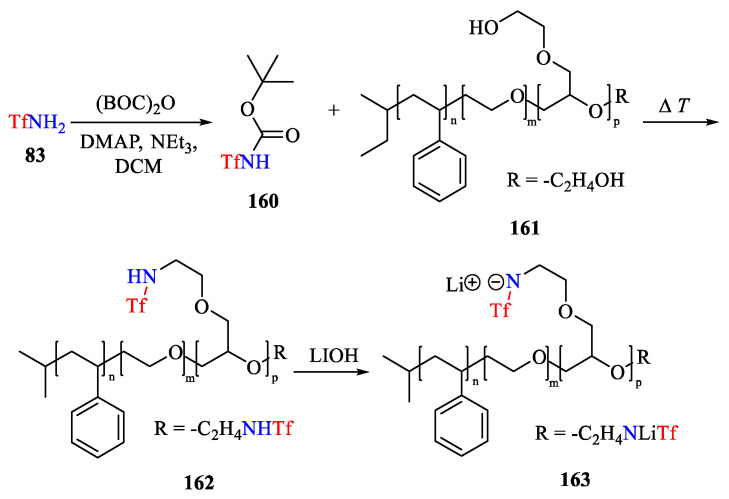
Synthesis of *tert*-butyl((trifluoromethyl)sulfonyl)carbamate (BTFSA) and synthesis route for the incorporation of TFSA groups at the polyether block.

Based on the triflamide derivatives, a new class of block of polystyrene-block-poly(ethylene oxide-co-hydroxyethyl glycidyl ether)diblock copolymers **163** is developed [153] (Scheme 45):

The resulting materials are currently being used to produce the next generation of single-ion-conducting polymer electrolytes (SICPEs) that are capable of competing with known Tf_2_NLi-based materials. In solid single-ion-conducting polymer electrolytes, the anion is covalently attached to the polymer and, ideally, only the cation contributes to the conductivity. The use of these electrolytes effectively eliminates many of the current safety and performance issues of the current liquid and salt-in-polymer electrolytes.

**Scheme 46:**
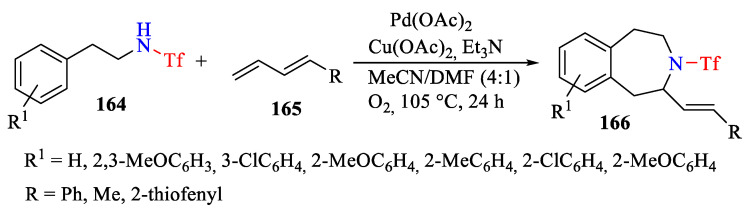
Reaction of Phenethyltriflamides with 1,3-Dienes to dopaminergic 3-Benzazepines.

*N-*Phenethylenetriflamides **164** react with 1,3-dienes **166** in the presence of catalytic amounts of Pd(OAc)_2_ and Cu(OAc)_2_/O_2_ as an oxidizing agent [154] (Scheme 46):

The reaction proceeds chemo-, regio- and diastereoselectively with the formation of 2,3,4,5-tetrahydro-*1H-*benzo[d]azepines **166**. The use of O_2_ as a co-oxidant makes it possible to reduce the amount of the oxidizing agent (Cu(OAc)_2_). 3-Benzazepines are present in a wide variety of natural products and important pharmaceuticals. These compounds are among the most convenient in terms of structural proximity and selectivity for dopamine D1 receptors, which regulate cell growth and development. As drugs for the treatment of CNS diseases, dopaminergic 3-benzazepines have the selective agonist or antagonist properties of dopamine D1 receptors, which have led to the development of pharmaceutical drugs against Parkinson’s disease, leukemia, cocaine addiction and obesity [154]. Triflamide derivatives **166** containing a 3-benzazepine moiety can be used to treat Alzheimer’s disease [155]. It should be noted that the synthesis of the *N-*alkyl-substituted triflamides presented in the scheme above is carried out by the reaction of TfNH_2_ with the corresponding alcohol in the presence of Cs_2_CO_3_ and an Ir-centered catalyst [156]. Such substituted sulfonamides exhibit a number of different types of biological activity (e.g., anti-inflammatory activity) [156].

**Scheme 47:**
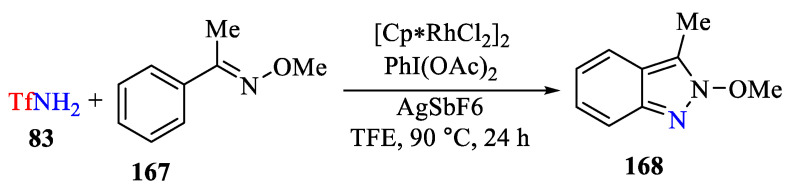
Catalyzed oxidative annulation of ketoximes with sulfonamide.

With the participation of triflamide **83**, the rhodium(III)-catalyzed intermolecular reaction of the C–H amination of ketoxime **167** in the presence of iodobenzene diacetate takes place [157] (Scheme 47):

In this case, under the conditions of an oxidative reaction, triflamide acts as a source of nitrogen during amination. The product **168** is formed in a 51% yield [157].

**Scheme 48:**
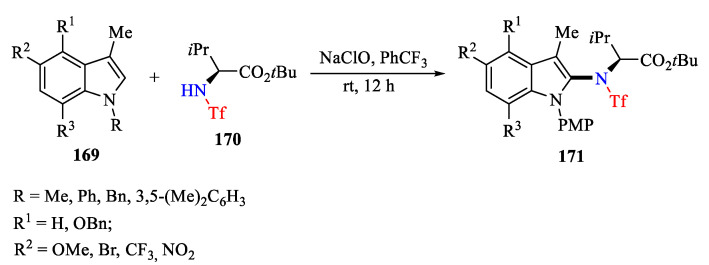
NaClO-promoted amination of 3-substituted indoles with TfNH-containg amino acid derivatives.

In the presence of NaOCl, the C–H amination reaction of 3-substituted indoles **169** with chiral triflamide-containing amino acids **170** proceeds. A feature of the reaction is the formation of a new class of atropisomers **171** [158] (Scheme 48):

The yield of the product **171** is 50–92%. Indoles are among the most important heteroarenes that are present in natural products and pharmaceuticals. The compounds are of great interest in the field of synthetic and medicinal chemistry [158].

**Scheme 49:**
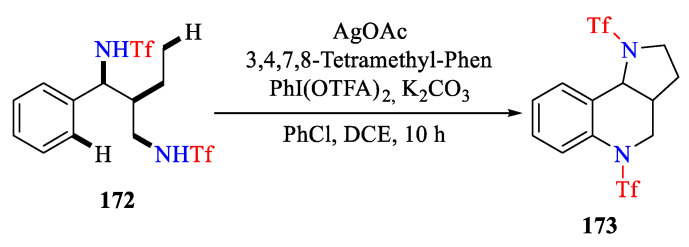
Silver-catalyzed direct amination of unactivated C–H bonds.

A unique property of triflamide derivatives is the ability to aminate non-activated C-H bonds, which is a great advantage in the synthesis of *N-*containing molecules due to the high efficiency and atom economy of such methods. A very unusual reaction of selective intramolecular amination at the C–H bond in the presence of silver salts resulting in functionalized heterocyclic products was published [159] (Scheme 49):

The method is effective for the transformation of non-functionalized groups in organic molecules and for the creation of complex structural units in natural and bioactive molecules; the reaction is most effective in the amination of primary sp*^3^* carbon atoms as the reactions proceed chemo- and regioselectively in comparison with the existing methods of direct amination (the Hoffmann–Löffler–Freitag reaction and the nitrene insertion reaction) [159].

**Scheme 50:**
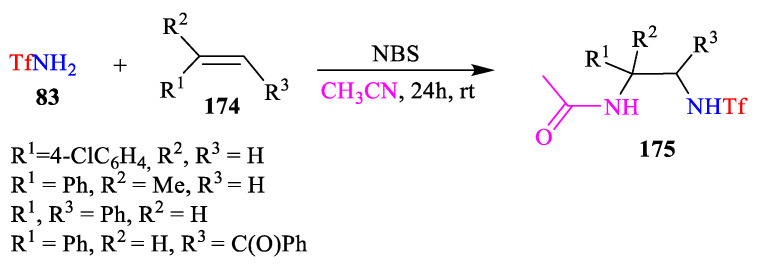
Amination of styrenes with TfNH_2_ in the presence of NBS.

Over the past few years, the reactions of triflamide with alkenes and dienes in the presence of oxidizing agents have been thoroughly studied in our group. Triflamide **83** reacts with styrene derivatives **174** and NBS in acetonitrile to form a product **175** with two different amine functional groups, *N*-trifluoromethanesulfonyl and acetamide. Obviously, solvent molecules (CH_3_CN) are involved in the reaction in this case [160] (Scheme 50):

Product yields are 76–94%.

**Scheme 51:**
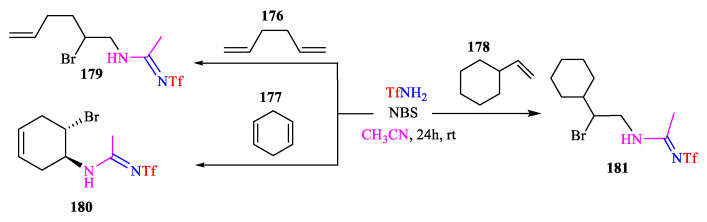
Amination of vinylcyclohexane, hexa-1,5-diene and cyclohexa-1,4-diene with TfNH_2_ in the presence of NBS.

**Scheme 52:**
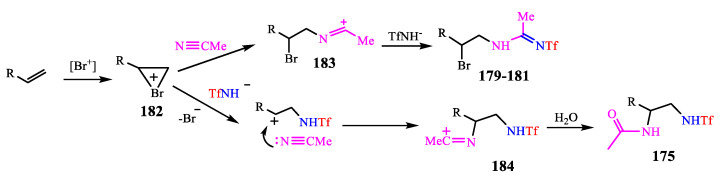
Formation of amidines or acetamides from alkenes and TfNH_2_ in the presence of NBS.

**Scheme 53:**
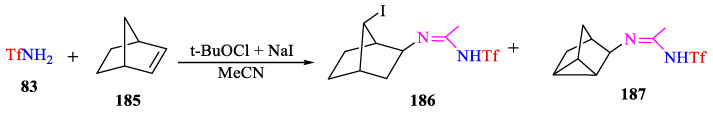
Reaction of norbornene with TfNH_2_ in the presence of t-BuOCl + NaI in CH_3_CN.

With alkenes **176**–**178** that do not have aryl groups, the reaction proceeds with the formation of *N-*sulfonylamidines **179**–**181** in quantitative yields [160] (Scheme 51):

Amidines **179**–**181** and acetamides **175** can be formed independently from the common intermediate bromonium ion **182**, which could be opened via either the CH_2_–Br or CH–Br bond splitting, leading, respectively, to amidines **179**–**181** or the products of hydrolysis (acetamides) **175** [160] (Scheme 52):

Unexpectedly, triflamide **83** reacts with norbornene **185** in acetonitrile with the insertion of the solvent and skeletal rearrangement, leading to the formation of iodine-containing acetamidine **186** and the tricyclic product **187** [161] (Scheme 53):

If NBS or NIS are used as the oxidizing agent, only halogen-amidines are formed, similar to those presented in Scheme 51 [161].

**Scheme 54:**
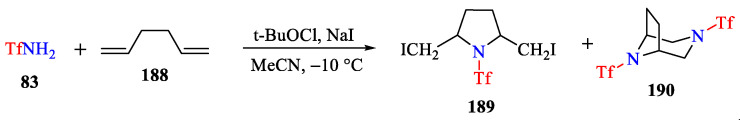
Reaction of 1,5-hexadiene **188** with TfNH_2_ **83** in the presence of t-BuOCl + NaI in CH_3_CN.

**Scheme 55:**
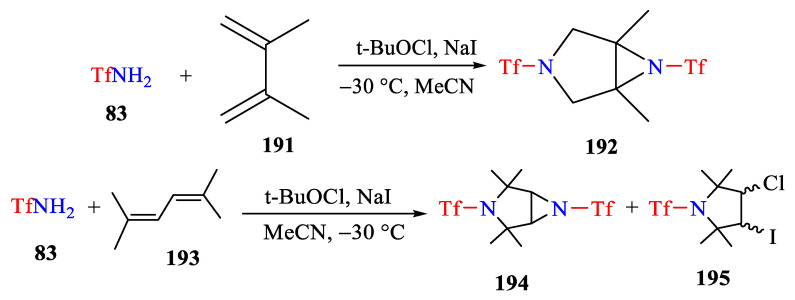
Heterocyclization of 2,3-dimethylbuta-1,3-diene and 2,5-dimethylhexa-2,4-diene. with TfNH_2_ in the presence of t-BuOCl + NaI in CH_3_CN.

The reaction of triflamide **83** with 1,5-hexadiene **188** was also studied. Two products were obtained in the total isolated yield of 91%; the corresponding pyrrolidine **189** and 3,8-bis(trifluoromethanesulfonyl)-3,8-diazabicyclo[3.2.1]octane **190** formed in the ratio of 3:2 [162] (Scheme 54):

We have also proposed a simple one-pot synthesis of the 3,6-diazabicyclo[3.1.0]hexane framework based on the reaction of triflamide **83** with 2,3-dimethylbuta-1,3-diene **191** and 2,5-dimethylhexa-2,4-diene **193** in the presence of the *t-*BuOCl + NaI system [163]. The reaction with 2,3-dimethylbuta-1,3-diene **191** leads to a single product **192** in an 80% yield (Scheme 55):

A similar reaction of triflamide with 2,5-dimethylhexa-2,4-diene **193** carried out at –30 °C afforded the corresponding bicyclic product **194** in a 37% yield and a 1:1 mixture of pyrrolidine diastereomers **195** in a 54% yield. The mechanism of the reaction of triflamide **83** with dienes **191** or **193** is supposedly heterocyclization to 3-pyrrolines and the subsequent aziridination of 3-pyrrolines and results in 3,6-diazabicyclo[3.1.0]hexanes **192** or **194**. Pyrrolidines **195** in this case are formed by the nucleophilic attack of the aziridine ring by Cl¯ or I¯ anions, presented in the reaction mixture, which is accompanied by the opening of the aziridine ring in the bicyclic product [163].

## 6. Conclusions

To summarize, triflimide is a unique reagent in organic synthesis. Possessing strong acidity and low nucleophilicity, it is successfully used for the design of new organic molecules, making it possible to form new C–C and C–heteroatom bonds. Given the low electron density of the nitrogen atom in Tf_2_NH and the steric hindrances around the nitrogen atom, it is a weak nucleophile for addition reactions. Due to these properties, the Tf_2_N¯ ion is actively used as a harmless counterion for cationic catalysts based on various metals to create substances that are important from a biological, pharmaceutical and industrial point of view. It is the ease of use, small loads and mild reaction conditions that make it possible to use triflimide both as a direct reagent in various reactions and as a catalyst/co-catalyst. Triflimide itself, as well as its derivatives, are effective in cascade cyclizations, cycloaddition, olefination, iodination, amination, etc. All these reactions lead to a huge number of new synthetically and biologically important objects, which undoubtedly make a significant contribution not only to fundamental organic chemistry but also in many additional applications.

Some triflamides demonstrate high anti-diabetic activity, high cytotoxicity for human cancer cell lines, anti-mycobacterial activity and anti-HIV activity. Triflamide derivatives are used as non-steroidal anti-inflammatory and anti-viral drugs, drugs for the treatment of hyperuricemia and effective drugs in the treatment of the female reproductive system diseases.

The introduction of a triflamide in aryl-containing molecules makes the modification of such substrates for biocatalytic hydroxylation possible; some triflamide compounds possess extraordinary antioxidant activity. Various triflamide compounds are widely used as peptidomimetics, organic catalysts showing high stereoselectivity and asymmetric transformations. The oxidative addition of triflamide to unsaturated substrates is a convenient method for amination and further heterocyclization under mild conditions.

Triflamides in all their various forms have certainly influenced most areas of synthetic organic chemistry in the last 10 years. Special physical and chemical properties of triflamides are used very widely, especially in medicine and pharmaceuticals for the development and synthesis of drugs and prodrugs containing the triflamide group as a key structural unit that determines biological activity, which will certainly be widely used in the future. In addition, in recent years there has been a growing interest in the use of triflamides as powerful electrophiles in a wide range of reactions. Triflamides are used as a hydrophobic solvent in lithium–sulfur batteries. It can be easily argued that the most promising field of application of triflamides are electrochemical energy storage systems. Triflamides can greatly influence the reactivity of chemical reactions compared to alkyl- and arylsulfonamides. Thus, this modifying effect will be widely used in synthetic organic chemistry and catalysis.

## Data Availability

Not applicable.

## Sample Availability

Samples of the compounds are available from the authors.

## Abbreviations

DMEDA	1,2-Dimethylethylenediamine
DMAP	4-Dimethylaminopyridine
HBTU	Hexafluorophosphate Benzotriazole Tetramethyl Uronium
EDCl	EDC-1-Ethyl-3-(3-dimethylaminopropyl)carbodiimide
HOBt	Hydroxybenzotriazole
DIH	1,3-Diiodo-5,5-Dimethylhydantoin
dF-CH3-ppy	2-(2,4-difluorophenyl)-5-methylpyridine
dtbbpy	4,4′-Di-tert-butyl-2,2′-dipyridyl
TTBP	Tri-Tert-Butylphenol
TFE	Trifluoroethanol
Phen	phenethylamine
DCE	1,2-Dichloroethane
HFIP	Hexafluoroisopropanol
XPhos	Dicyclohexyl[2′,4′,6′-tris(propan-2-yl)[1,1′-biphenyl]-2-yl]phosphane
DCM	Dichloromethane
TEA	triethanolamine
Dppf	1,1′-Bis(diphenylphosphino)ferrocene
Bpy	2,2′-Bipyridine
